# HIV-1 Envelope Glycoprotein Resistance to Monoclonal Antibody 2G12 Is Subject-Specific and Context-Dependent in Macaques and Humans

**DOI:** 10.1371/journal.pone.0075277

**Published:** 2013-09-09

**Authors:** Delphine C. Malherbe, Rogier W. Sanders, Marit J. van Gils, Byung Park, Michelle M. Gomes, Hanneke Schuitemaker, Susan Barnett, Nancy L. Haigwood

**Affiliations:** 1 Oregon National Primate Research Center, Oregon Health and Science University, Beaverton, Oregon, United States of America; 2 Laboratory of Experimental Virology, Department of Medical Microbiology, Center for Infection and Immunity Amsterdam, Academic Medical Center of the University of Amsterdam, Amsterdam, The Netherlands; 3 Department of Microbiology and Immunology, Weill Medical College of Cornell University, New York, New York, United States of America; 4 Department of Public Health and Preventive Medicine, Oregon Health and Science University, Portland, Oregon, United States of America; 5 Laboratory of Viral Immune Pathogenesis, Department of Experimental Immunology, Center for Infection and Immunity Amsterdam, Academic Medical Center of the University of Amsterdam, Amsterdam, The Netherlands; 6 Crucell, Leiden, The Netherlands; 7 Novartis Institutes for Biomedical Research, Cambridge, Massachusetts, United States of America; University of Missouri, United States of America

## Abstract

HIV-1 Envelope (Env) protein is the sole target of neutralizing antibodies (NAbs) that arise during infection to neutralize autologous variants. Under this immune pressure, HIV escape variants are continuously selected and over the course of infection Env becomes more neutralization resistant. Many common alterations are known to affect sensitivity to NAbs, including residues encoding potential N-linked glycosylation sites (PNGS). Knowledge of Env motifs associated with neutralization resistance is valuable for the design of an effective Env-based vaccine so we characterized Envs isolated longitudinally from a SHIV_SF162P4_ infected macaque for sensitivity to neutralizing monoclonal antibodies (MAbs) B12, 2G12, 4E10 and 2F5. The early Env, isolated from plasma at day 56 after infection, was the most sensitive and the late Env, from day 670, was the most resistant to MAbs. We identified four PNGS in these Envs that accumulated over time at positions 130, 139, 160 and 397. We determined that removal of these PNGS significantly increased neutralization sensitivity to 2G12, and conversely, we identified mutations by *in silico* analyses that contributed resistance to 2G12 neutralization. In order to expand our understanding of these PNGS, we analyzed Envs from clade B HIV-infected human subjects and identified additional glycan and amino acid changes that could affect neutralization by 2G12 in a context-dependent manner. Taken together, these *in vitro* and *in silico* analyses of clade B Envs revealed that 2G12 resistance is achieved by previously unrecognized PNGS substitutions in a context-dependent manner and by subject-specific pathways.

## Introduction

The HIV-1 envelope glycoprotein complex (Env) is the sole target of neutralizing antibodies (NAbs) and it evolves rapidly to escape this immune pressure. NAbs arise during infection to neutralize autologous variants and a limited number of HIV patients develop broad neutralizing antibodies [1,2]. There is continuous viral escape and selection by autologous NAbs [3,4] and recent studies identified multiple escape pathways varying from patient to patient and over the course of infection [5,6]. Escape mechanisms include: [a] amino acid sequence variation [7]; [b] entropic masking of Env [8]; [c] flexibility in size and positioning of the variable loops [9,10]; and [d] changes of glycosylation patterns [4,11,12]. Glycans are attached to the motif: N-X-S/T, (where X can be any amino acid except a proline) that defines Potential N-linked Glycosylation sites (PNGS). Over the course of infection, the locations of PNGS are altered [13] and the number of PNGS is increased [14]. Paradoxically, recent studies identified glycans as targets of the very potent PGT monoclonal antibodies (MAbs) [15,16].

Knowing how shifting PNGS as well as other mutations affect neutralization resistance could yield useful information for vaccine design. We recently showed that exposing rabbits sequentially to Env variants collected over a 2-year period from a SHIV_SF162P4_-infected macaque (A141) with a moderate cross-NAb response, better educated the immune system to elicit a cross-reactive response [17]. In the current study, we further defined the Env variants isolated from this SHIV-infected macaque and identified a set of four PNGS at positions 130, 139, 160 and 397 (respectively located in V1, V2 and V4) that increased neutralization resistance to MAbs, in particular 2G12. In contrast to other MAbs, 2G12 structure is unusual with swapped variable domains that enable recognition of glycans on the silent face of gp120 [18]. Crucial Env PNGS for 2G12 binding are N295 (in C2) and N332 (in C3) while accessory PNGS are N339 (in C3), N386, N392, N397 (in V4) and N448 (in C4) [19,20]. Similar to our findings, a few recent studies identified other changes in Env variable loops that affect 2G12 neutralization in a subject-specific manner [21,22]. Our data also show that neutralization could also be affected by supplementary amino acid mutations located in V1/V2, C2, V3 and C3 regions. Furthermore, *in silico* studies on clade B Envs from human subjects revealed that other, distinct PNGS and amino acid changes mediated neutralization-resistance, thereby identifying new pathways leading to 2G12 resistance that are subject specific.

## Materials and Methods

### Envelope sequences and *in silico* analyses

The envelope nucleotide sequences used in this study are deposited in GenBank [17,23,24]. The nucleotide sequences were aligned to HxB2 sequence and translated into protein alignment using the HIValign tool (http://www.hiv.lanl.gov/content/sequence​/VIRALIGN/viralign.html). The PNGS analysis was performed on the protein alignment with the Aminotrack webserver (http://apps.sbri.org/AminoTrack/). The identification of other amino acid changes between envelopes was performed manually with the Geneious Pro software 5.4.6 (Biomatters, Auckland, New Zealand).

### Site-directed mutagenesis

The mutations of interest in A141 were introduced by site-directed mutagenesis with the QuikChange Multi Site-Directed Mutagenesis Kit (Stratagene, La Jolla, CA). Briefly, we introduced three sets of PNGS changes onto the d670 A8-9 backbone: Δ(N130/N397) to replicate d417 D15 PNGS profile (changes N130A and T399A), Δ(N139/N397) to replicate d487 A6-1 PNGS profile (changes N139A and T399A) and Δ(N130/N139/N160/N397) to replicate d56 B3 PNGS profile (changes N130A, N139A, N160A and T399A). The single PNGS mutant ∆N397 was also generated (T399A change). The PCR conditions were as follows: 1 min at 95°C, 30 x [1 min at 95°C, 1 min at 55°C, 12 min at 65°C]. The presence of the mutation of interest was verified by DNA sequencing using Prism dye terminator kits (ABI, Foster City, CA) on an Applied Biosystems 3730XL genetic analyzer. Nucleotide alignments were manually edited using the Sequencher software program (Gene Codes Corporation, Ann Arbor, MI).

### Pseudoviruses

Pseudoviruses were produced with the pSG3ΔEnv DNA plasmid encoding the HIV backbone and a plasmid encoding the envelope of interest. Briefly, 293T cells were plated at 5x10^5^ cells/well in 6-well plates in medium (DMEM, 10% fetal calf serum, 1% L-glutamine, 1% penicillin-streptomycin). The following day, 293T cells in DMEM were transfected with 1 µg total DNA/well at a 20:1 ratio of backbone to Env plasmids in the presence of 10.5 µg polyethylenimine (PEI; Polysciences, Inc., Warrington, PA) per well. Cells were incubated for 48 hours at 37°C. Virus was harvested by centrifugation of the supernatant (2,000 rpm for 10 min at 4°C) and stored at -80°C until use. The pseudovirus was titrated on Tzm-bl cells. Dilutions of the pseudovirus were used to infect TZM-bl cells in the presence of 7.5 µg/ml DEAE-dextran (Fisher Biotech, Fair Lawn, NJ). Two days later, infection was measured by luciferase expression and 200 50% tissue culture infective doses (TCID_50_) (determined by the Reed-Muench formula) were added to each neutralization assay.

### Neutralization assay

Monoclonal antibodies IgG1 b12 (gift of D. Burton, The Scripps Research Institute, La Jolla, CA), 2G12, 2F5 and 4E10 (POLYMUN Scientific GMBH, Vienna, Austria) were assessed for neutralization of the pseudoviruses bearing the envelope of interest in a TZM-bl neutralization assay as previously described [25]. Briefly, 200 TCID_50_ of virus were added to serial dilutions of monoclonal antibody and incubated in a total volume of 150 µl medium (DMEM, 10% fetal calf serum, 1% L-glutamine, 1% penicillin-streptomycin) for 1 h at 37°C before addition of 100 µL of Tzm-bl cells resuspended in medium at 1 x 10^5^ cells/mL in the presence of 7.5 µg/mL DEAE-dextran. Forty-eight hours later, 150 µL of medium was removed from each well and cells were lysed for 2 min directly in the neutralization plate, using 100 µL of Bright-Glo luciferase assay substrate (Promega, Madison, WI), and immediately analyzed for luciferase activity on a luminometer (Victor, PerkinElmer, Waltham, MA). All values were calculated with respect to virus only wells [(value for virus only minus cells only) minus (value for serum minus cells only)] divided by (value for virus minus cells only).

### Immune precipitation

Pseudovirus lysates of A141 Envs [d56 B3, d417 D15, d487 A6-1, d670 A8-9] and the different PNGS mutants [Δ(N130/N397), Δ(N139/N397), Δ(N130/N139/N160/N397) and ΔN397] as well as the Env-deficient pseudovirus (pSG3ΔEnv) were generated as described [26] and assessed for total protein content with a Bradford assay (Pierce, Rockford, IL). An equal protein amount was precipitated with 10 µg/mL 2G12 for 3 hrs at room temperature before overnight incubation at 4°C with protein G sepharose beads (GE Life Sciences, Piscataway, NJ). Samples were run on a SDS-PAGE and transferred to nitrocellulose prior to probing with rabbit anti-Env polyclonal serum as previously described [26].

### 3D modeling

A three-dimensional model of monomeric gp120 was built based on the structure of the HIV-1 JRFL gp120 core protein complexed with CD4 and the X5 antibody (PDB code 2B4C) [27] by homology modeling using the SWISS-MODEL modeling tool (http://swissmodel.expasy.org/) [28–30] and the input sequences of gp120 from SF162. Quality of the final model was evaluated using ANOLEA and GROMOS and found to be satisfactory [31]. The figure was prepared using PyMOL Molecular Graphics System, Version 1.2r1, Schrödinger, LLC [32].

### Statistical analyses

Permutation test was used to investigate the difference in IC_50_ between the neutralization of the pseudovirus/PNGS mutant of interest in a TZM-bl cell assay. For each monoclonal antibody, we randomly selected about 50,000 permutations from possible 1.82509x10^14^ distinct ways to assign 21 observations into 7 equal groups. One-way analysis of variance (ANOVA) was performed on each 50,000 permutation samples followed by 12 contrast comparisons. The permutation p-value for each comparison was obtained from more than 50,000 statistical hypothesis testing.

## Results

### Early variants are more sensitive to MAbs than late variants

**Figure 1:**
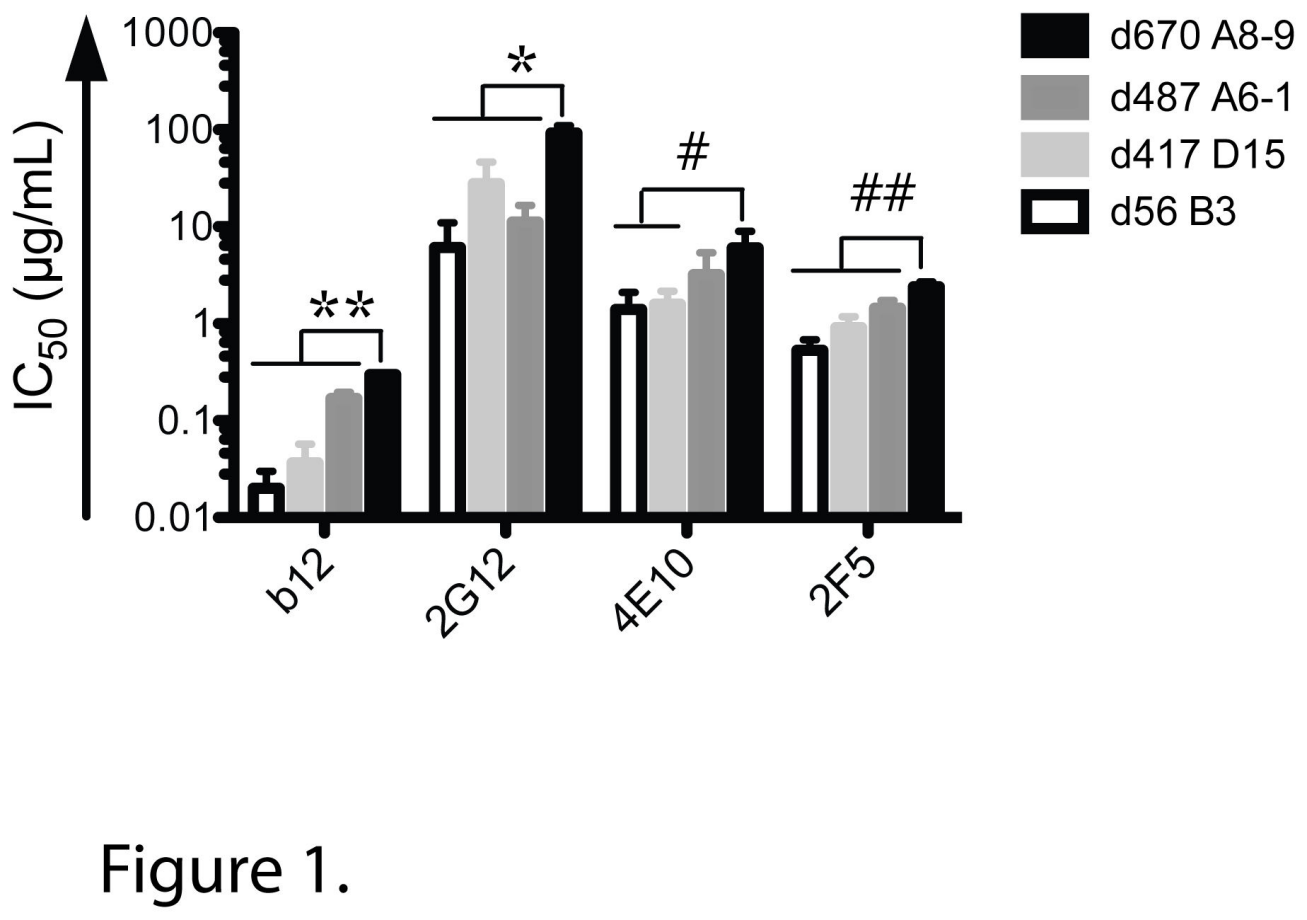
Early A141 variants are more sensitive to monoclonal antibodies than late variants. Serially diluted MAbs (b12, 2G12, 4E10 and 2F5) were tested for the neutralization of the pseudovirus of interest (d56 B3, d417 D15, d487 A6-1 and d670 A8-9) in a TZM-bl cell assay. The MAb concentration (µg/mL) required to reach 50% neutralization of each pseudovirus is reported as IC_50_ (µg/mL). Data shown are mean +/- SD from three independent assays. Compared to d670 A8-9: *P < 0.005, **P < 0.0005, ^#^ *P* < 0.02, ^# #^ *P* < 0.003.

Neutralization sensitivity to well-defined MAbs b12, 2G12, 2F5 and 4E10 was used to characterize different A141 Env variants (Figure 1). b12 recognizes an epitope that overlaps the CD4 binding site [33] and 2G12 targets glycosylation sites [19,34,35]; both epitopes are conformational and located in gp120. In contrast, 2F5 and 4E10 recognize adjacent linear epitopes in the membrane proximal external region (MPER) of gp41 [36,37]. The four envelope glycoproteins tested here were isolated longitudinally from A141, a SHIV_SF162P4_-infected macaque that developed moderate breadth [38]: d56 B3, an early variant isolated 56 days post-infection (dpi); d417 D15 and d487 A6-1, two intermediate clones isolated 417 and 487 dpi; and d670 A8-9 a late variant isolated 670 dpi. The early Envelope d56 B3 was the most sensitive to all four tested MAbs and the late Envelope d670 A8-9 was the most resistant (Figure 1). The two middle envelope glycoproteins d417 D15 and d487 A6-1 displayed intermediate sensitivity phenotypes. d670 A8-9 Envelope was significantly more difficult to neutralize by all MAbs tested and was particularly resistant to 2G12. As previously seen in some HIV-infected human subjects [23], these results show that over the course of infection in macaques, HIV-1 Env evolves to become more resistant to neutralization by MAbs that target certain conserved determinants.

### Role of specific PNGS in neutralization resistance

**Figure 2:**
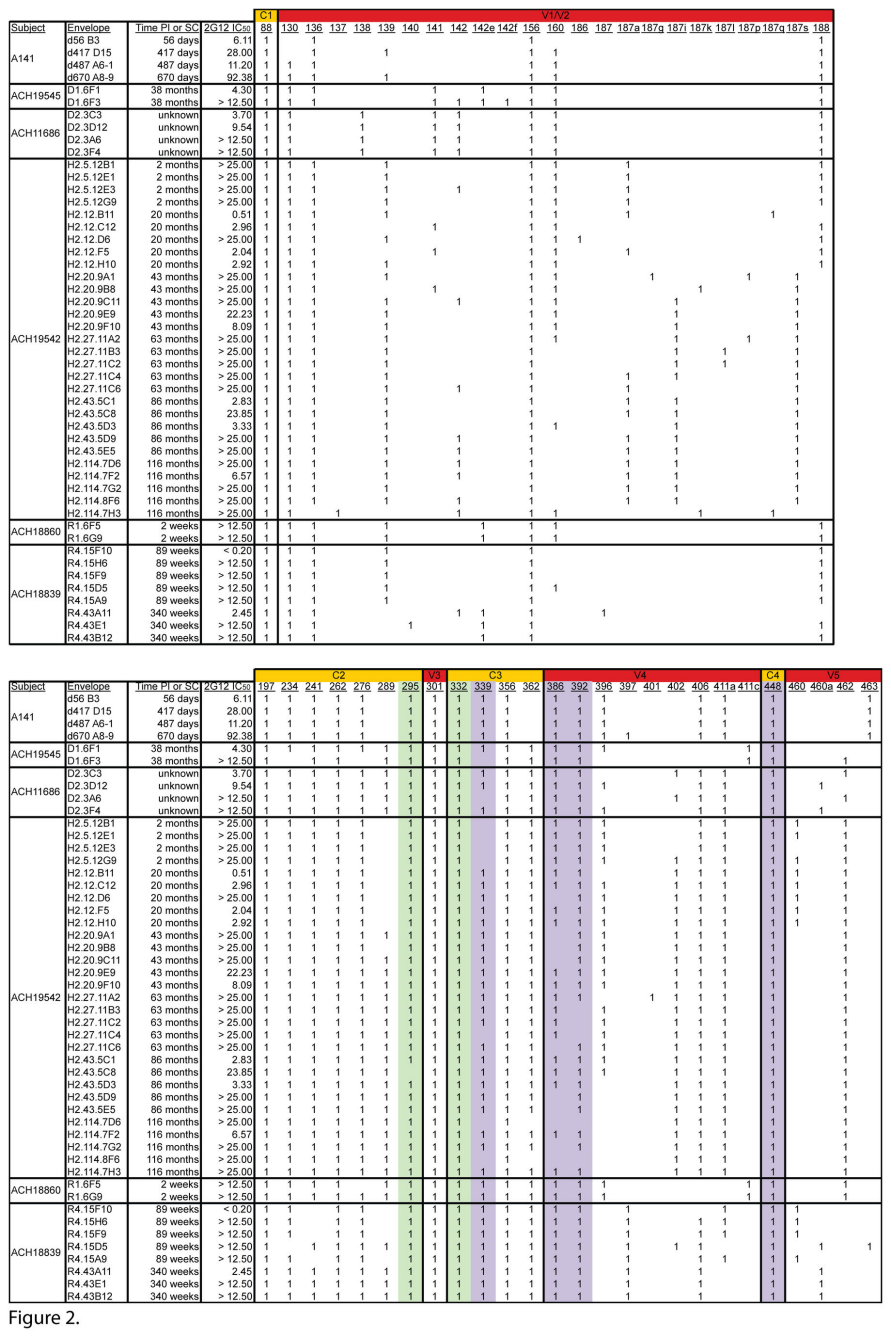
PNGS changes in different clade B Env quasispecies. Regions of Envelope are noted in the top bar and below are the PNGS positions. All protein sequences were aligned to HXB2 (accession # AF033819) and were analyzed with the Aminotrack webserver (http://apps.sbri.org/AminoTrack/). 1: Presence of PNGS at a given amino acid position. Green boxes indicate the essential PNGS and purple boxes indicate the accessory PNGS for 2G12 recognition. 2G12 IC_50_ (µg/mL) and the time post infection (PI) for the envelopes from the A141 macaque are indicated. Neutralization data and time since seroconversion (SC) for subjects ACH19545, ACH11686, ACH 18860 and ACH18839 from the Amsterdam cohort are reported per Quakkelaar et al. [24]. 2G12 IC_50_ (µg/mL) and time since SC for subject ACH19542 from the Amsterdam cohort are adapted from Bunnik et al. [23]..

**Figure 3:**
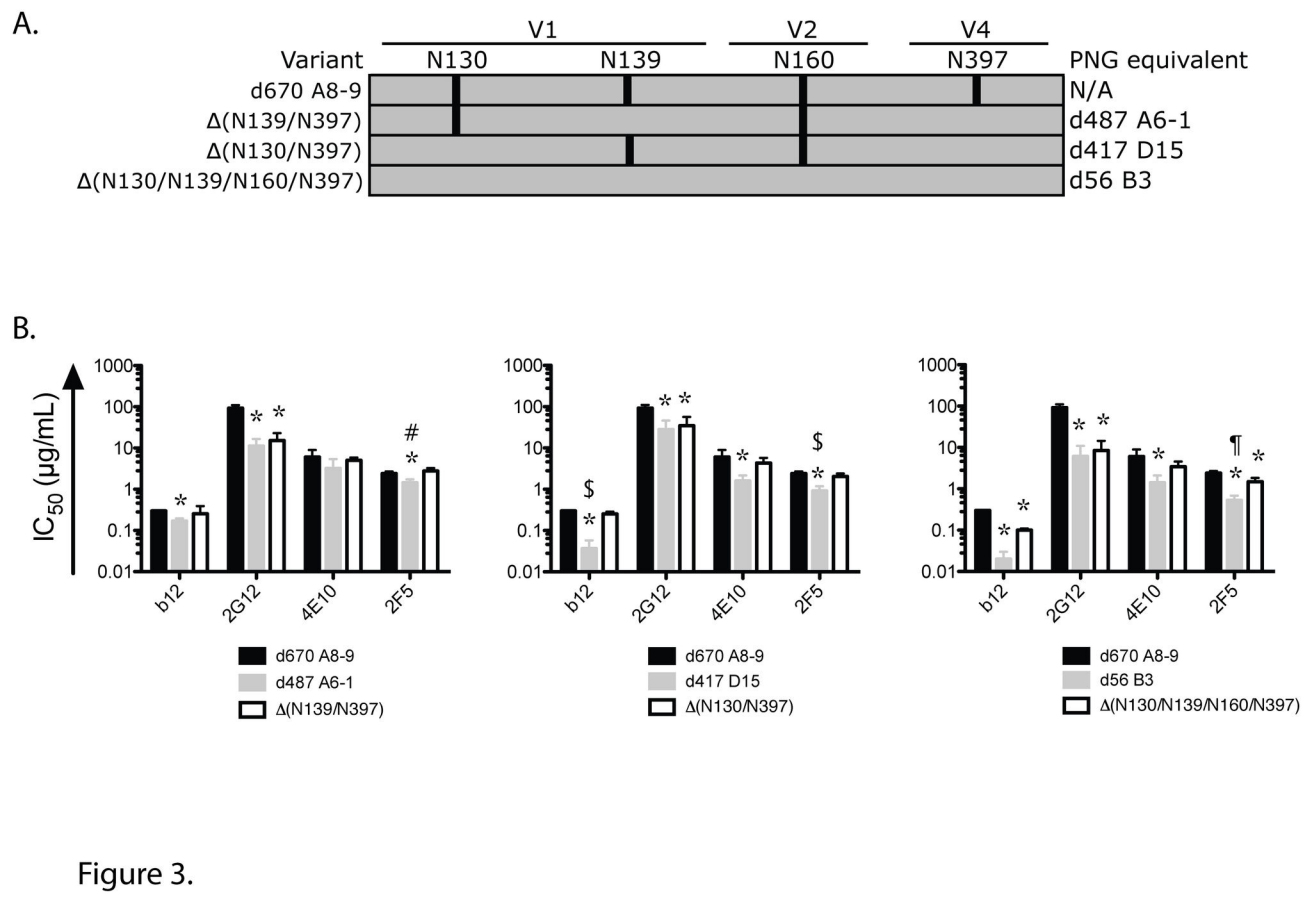
Specific PNGS affect heterologous neutralization. (**A**) Cartoon depicting PNGS profiles of A141 Env mutants. The mutants are indicated on the left and the corresponding PNG equivalent A141 clones are indicated on the right. Vertical bars indicate the presence of a PNGS at the amino acid positions (130, 139, 160 or 397) shown. (**B**) Serially diluted monoclonal antibodies (b12, 2G12, 4E10 and 2F5) were tested for the neutralization of the pseudovirus of interest [d56 B3, d417 D15, d487 A6-1 and d670 A8-9] or of the PNG mutant of interest [Δ(N130/397), Δ(N139/397) and Δ(N130/139/160/397)] in a TZM-bl cell assay. The MAb concentration (µg/mL) required to reach 50% neutralization of each pseudovirus is reported as IC_50_ (µg/mL). Data shown are mean +/- SD from three independent assays. *P* < 0.02 compared to d670 A8-9. ^#^ *P* = 0.0003 compared to d487 A6-1. ^$^ *P* ≤ 0.0006 compared to d417 D15. ^¶^ *P* = 0.0042 compared to d56 B3.

**Figure 4:**
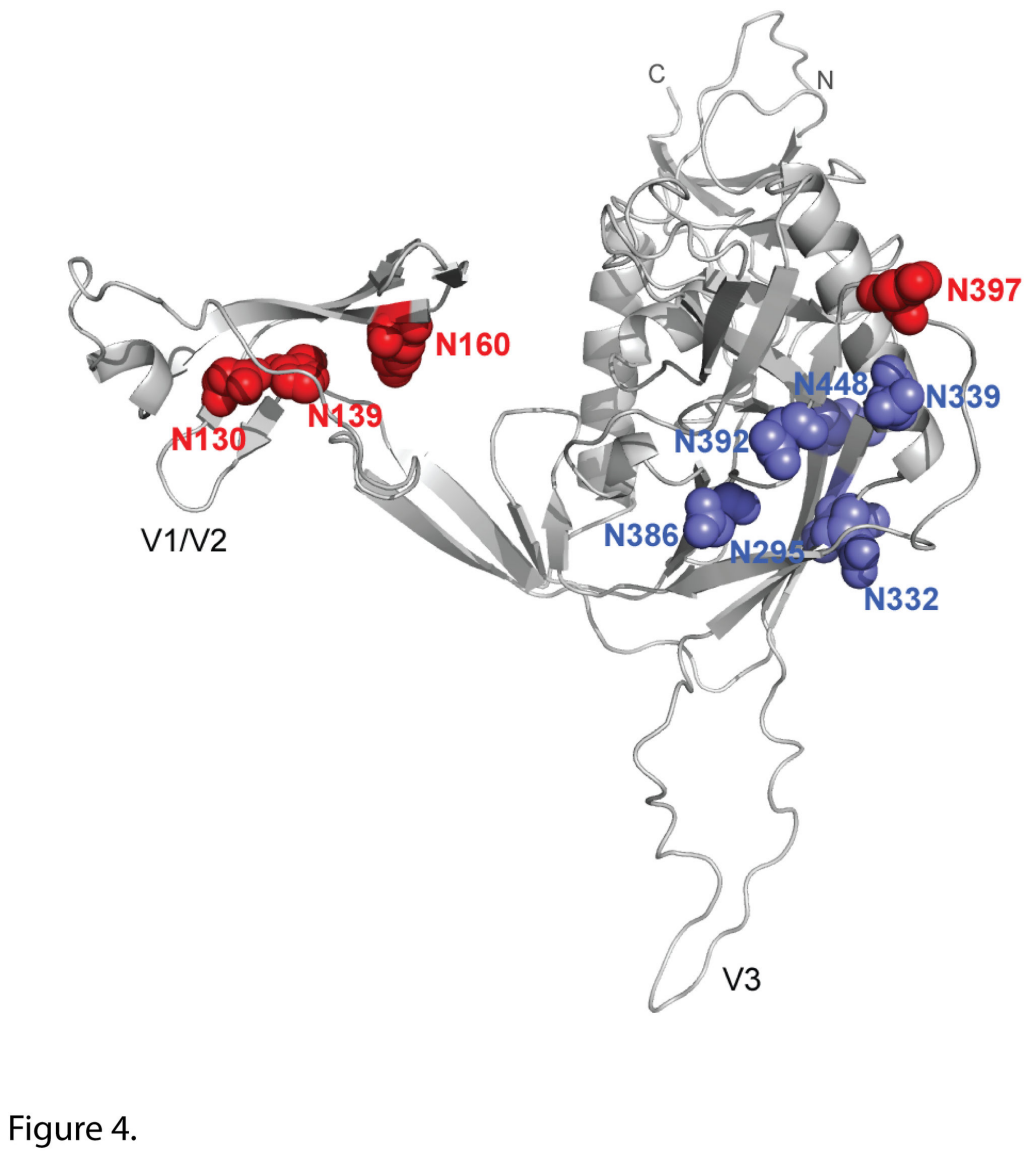
3D model of PNGS of interest for 2G12 recognition. The three-dimensional model of SF162 gp120 monomer (light grey) shows the location of the four PNGS of interest (red spheres) and the N-glycans (blue spheres) important for 2G12 binding. The model was built by homology modeling using the SWISS- MODEL server (http://swissmodel.expasy.org/) based on the structure of the HIV-1 JRFL gp120 core protein complexed with CD4 and the X5 antibody (PDB code 2B4C) [27]. The figure was generated with PyMOL Molecular Graphics System.

Given the neutralization data obtained with the different MAbs, especially 2G12 (Figure 1), we investigated whether the difference in neutralization could be correlated with differences in specific PNGS. PNGS N295 and N332 are crucial to 2G12 binding, and other PNGS are marginally involved including N339, N386, N392, N397 and N448 [19,20]. All the A141 variants have PNGS N295 and N332 (Figure 2). In addition, all possess PNGS N339, N386, N392 and N448 whereas only d670 A8-9 has N397. Therefore, neutralization resistance to 2G12 could not be explained by the presence or absence of the specific PNGS involved in 2G12 epitope. Indeed, removal of PNGS N397 from d670 A8-9 did not affect neutralization by 2G12 or the other three MAbs assessed in this study (data not shown). Instead, a comparison of PNGS prevalence revealed that N130 (V1), N139 (V1), N160 (V2) and N397 (V4) are present in different combinations in later clones that are more neutralization resistant but are absent in the early and neutralization sensitive clone d56 B3 (Figure 2). Indeed, all four PNGS are present in d670 A8-9 whereas d417 D15 has N139 and N160; and d487 A6-1 has N130 and N160. Therefore, we hypothesized that combinations of these four PNGS at positions 130, 139, 160 and 397 could affect neutralization sensitivity to 2G12 in the studied variants. We removed the PNGS by site directed mutagenesis from the d670 A8-9 backbone in order to replicate the observed PNGS profiles in d56 B3, d417 D15 and d487 A6-1 (Figure 3A). The A8-9 PNGS mutants that were created were functional in making virions that were infectious and capable of binding to 2G12. All naturally occurring A141 Envs [d56 B3, d417 D15, d487 A6-1, d670 A8-9] and the different PNGS mutants [Δ(N130/N397), Δ(N139/N397), Δ(N130/N139/N160/N397) and ΔN397], as well as the Env-deficient pseudovirus (pSG3ΔEnv) were tested in two independent experiments. 2G12 was able to precipitate all A141 and PNGS mutants pseudoviruses but not the Env-deficient virus, thereby showing that the interaction between the Envs described in this study and 2G12 is a direct interaction (data not shown). We then assessed the neutralization of these PNGS mutants by MAbs b12, 2G12, 2F5 and 4E10 (Figure 3B) and found that removal of the PNGS significantly enhanced sensitivity of all mutants to 2G12 compared to d670 A8-9 (*P* = 0.0003 with Δ(N139/N397), *P* = 0.0014 with Δ(N130/N397) and *P* = 0.0001 with Δ(N130/N139/N160/N397)). In addition the 2G12 IC_50_ obtained for the mutants was not statistically different from the IC_50_ obtained for the model clone (*P* = 0.84, Δ(N130/N139/N160/N397) versus d56 B3, *P* = 0.55, Δ(N130/N397) versus d417 D15 and *P* = 0.72, Δ(N139/N397) versus d487 A6-1, respectively) thus suggesting that only these PNGS affected 2G12 neutralization and not the d670 A8-9 backbone. Therefore, we identified previously unrecognized PNGS substitutions outside the 2G12 epitope [19,39] that affect 2G12 neutralization and we visualized them in a 3D model of gp120 Env (Figure 4).

Next, we compared the neutralization of the PNGS mutants by other MAbs (Figure 3B). The neutralization of these mutants by 4E10 was not statistically different from the one measured for d670 A8-9. In contrast, 2F5 displayed an intermediate neutralization level of the PNGS mutants (*P* = 0.0036 between Δ(N130/N139/N160/N397) and d56 B3, *P* = 0.0006 between Δ(N130/N397) and d417 D15 and *P* = 0.0003 between Δ(N139/N397) and d487 A6-1, respectively). In addition, Δ(N130/N397) displayed an intermediate neutralization phenotype by b12 (*P* = 0.0003 compared to d417 D15) thereby showing that other factors outside the studied PNGS also affect neutralization sensitivity. Overall, our results suggest that the elimination of four specific PNGS in gp120 can alter the conformation and/or accessibility of antibody epitopes on both gp120 and gp41.

### Substitution of positively charged residues with neutral or negatively charged residues in neutralization resistant variants

To identify additional features of these Envs affecting neutralization sensitivity, we assessed the possible participation of specific amino acids besides those involved in PNGS. A comparison of the four A141 Env sequences identified eight amino acid (AA) positions which were mutated in the late variants but not in the early one (Table 1). These positions are located in V1 (AA 141 and 151), in V2 (AA 160 and 191), in C2 (AA 281), in V3 (AA 315) and in C3 (AA 348 and 354). AA 141 is located in a hot spot for PNG variation as defined by Blay et al. [13], and in the late variants the mutation at that position induced a change in amino acid charge. The R151G and the K160N mutations are found in all three late variants where they induce a charge change; in addition K160N creates a PNGS. The K192N mutation induces a change in amino acid charge in d417 D15 and d487 A6-1 but it does not create a PNGS. A281 is located in C2 and is involved in the CD4 binding site [40] whereas R315 is part of the GPGR motif at the tip of V3 crown, which is the epitope for MAb 447-52D [41], and is also part of the epitope of MAb F425-B4e8 [42]. The three late variants harbor the A281V and R315K mutations and no charge change occurred at these positions. K348E and G354R are also found in all three late variants but they induce charge changes. Overall, all these amino acid mutations throughout Env substitute residues with positively charged side chains with residues with neutral or negatively charged chains.

**Table 1:** A141 Residues with positively charged side chains substituted for residues with neutral, negatively charged chains.

	**V1/V2**				**C2**	**V3**	**C3**		**2G12 IC_50_ (µg/mL)**
**AAposition**	**141**	**151**	**160**	**192**	**281**	**315**	**348**	**354**	
d56 B3	K*	R*	K*	K*	A	R*	K*	G	6.11
d417 D15	T	G	N	N	V	K*	E**	R*	28.00
d487 A6-1	E**	G	N	N	V	K*	E**	R*	11.21
d670 A8-9	T	G	N	R*	V	K*	E**	R*	92.38

Regions of Envelope are noted in the top bar and below are the amino acid (AA) positions. Each row is an Env clone. * Positively and ** negatively charged amino acids are in bold. 2G12 IC_50_ (µg/mL) is specified for each clone.

### 2G12 resistance is due to subject specific mutations

To further characterize the amino acid changes responsible for 2G12 resistance, we performed *in silico* analyses of clade B Envs from five Amsterdam Cohort human subjects [23,24], including some longitudinally-derived Envelopes (Figure 2). The PNGS combination responsible for 2G12 resistance in the A141 Envelopes was not present in these subjects (except Env ACH18839. R4.15D5). However some changes at positions 139 and 160 as well as other PNGS changes were observed. Importantly, some of the 2G12 resistance observed in ACH19545, ACH11686, ACH18860 and ACH19542 is most likely due to the absence of accessory PNGS for 2G12 interaction such as N397 in ACH18860; N339 and N397 in ACH19545; N339, N386 and N397 in ACH11686 and ACH19542. Altered PNGS at positions 139, 160 and others were observed in subject ACH18839, who otherwise possessed all required and accessory PNGS for 2G12 recognition. Interestingly, like d670 A8-9, 2G12-resistant ACH18839 Envs added PNGS at positions 397 and 406, but these PNGS are absent in ACH18860 2G12-resistant Envs.

An *in silico* analysis of the amino acid sequence revealed additional positions where mutations occurred between the 2G12 sensitive and 2G12 resistant clones in the ACH18839 Envelopes (Table 2). Similar to the A141 Envelopes, changes happened at positions 151 and 160 however all other mutations occurred at different positions in ACH18839 and A141. Charge changes took place at positions 151, 160, 336, 410, 412 and 463. Overall these mutations rendered envelope more negatively charged. Taken together these results show that different combinations of PNGS changes and amino acid changes occurred in these HIV-infected subjects and that the mutations that led to 2G12 resistance were subject specific.

**Table 2:** Residues in clones from subject ACH18839 with positively charged side chains substituted for residues with neutral, negatively charged chains.

	**V1/V2**			**C3**			**V4**					**V5**	**2G12 IC_50_**
**AAposition**	**151**	**160**	**187**	**336**	**343**	**347**	**407**	**408**	**409**	**410**	**412**	**463**	
R4.15F10	__	E**	G	K*	R*	D**	T	L	L	__	__	G	< 0.20
R4.43A11	K*	N	N	E**	K*	V	N	N	T	E**	E**	A	2.45
R4.15H6	K*	M	D**	E**	K*	N	N	T	T	E**	G	E**	> 12.50
R4.15F9	K*	M	D**	V	K*	A	N	T	T	E**	E**	E**	> 12.50
R4.15D5	G	N	D**	T	Q	K*	__	T	T	E**	__	S	> 12.50
R4.15A9	K*	M	D**	E**	K*	N	N	T	T	E**	G	E**	> 12.50
R4.43E1	K*	N	N	E**	K*	V	N	N	T	E**	E**	A	> 12.50
R4.43B12	K*	N	N	E**	K*	V	N	N	T	E**	E**	A	> 12.50

Regions of Envelope are noted in the top bar and below are the amino acid (AA) positions. Each row is an Env clone. * Positively and ** negatively charged amino acids are in bold. 2G12 IC_50_ (µg/mL) is specified for each clone.

## Discussion

HIV Envelope evolves under immune pressure and escape mechanisms include increase in variable loop length, introduction of specific amino acid mutations and additions and/or shift of potential N-linked glycosylation sites [43]. The current study identifies a previously unknown combination of four PNGS (located at positions 130, 139, 160 and 397) as a key motif affecting 2G12 neutralization resistance in Envelopes isolated from a SHIV-infected macaque. Importantly, investigation of clade B infected human subjects showed that resistance to MAb 2G12 is subject-specific and context-dependent in macaques and humans.

Our results identified previously four unknown PNGS at positions 130 (V1), 139 (V1), 160 (V2) and 397 (V4) that affect 2G12 neutralization and suggest that eliminating these specific PNGS modified Env conformation and/or access to gp120 and gp41. Similar to our observations with the late variant d670 A8-9, Nakowitsch identified 2G12-resistant viruses [44] that harbored all the PNGS required for 2G12 recognition [19,20]. Our data show that removing the four PNGS of interest from the d670 A8-9 backbone significantly enhanced sensitivity to 2G12 by all PNGS mutants, thereby demonstrating that 2G12 neutralization can be affected by these glycosylation changes located outside its epitope [19,35,39]. In addition, these findings are not restricted to clade B Envelopes since PNGS N442 also affected 2G12 neutralization in clade C Envs [45] despite being located outside the 2G12 epitope. Interestingly, we also found that the removal of the four PNGS affected neutralization by other MAbs. For example, the neutralization of several PNGS mutants by 2F5 and b12 was different from the one obtained with d670 A8-9 even though all variants possess the canonical 2F5 ELDKWA epitope and the main amino acids involved in binding to b12 [33]. Furthermore, the PNGS changes affecting b12 binding [46] are not found in these Envs. However, we cannot exclude that amino acid mutations in other parts of Env can affect the b12 epitope since in addition to the four PNG differences there are other amino acid changes between these four Envelopes. Indeed previous studies indicated that b12 neutralization is affected by steric constraints that limit b12 access to its epitope [47]. Similar findings were observed for 4E10 and 2F5 where PNGS changes at position 295 in SF162 and in clade C Envs increased neutralization sensitivity [45,48].

Our analysis of A141 Envelopes also shows that the late variants accumulate amino acid changes that could alter Env conformation due to different charges or that can directly affect neutralization resistance. Negative charge changes were introduced in V1 (AA 141, 151), V2 (AA 160) and C3 (AA 348, 354), which render the sequences more acidic. It has been proposed that this evolution in V1V2 may affect Env conformation in the vicinity of the CD4 binding site [13]. Interestingly, Ringe observed that positively charged residues in V2 enhanced neutralization sensitivity to the very potent MAbs PG9 and PG16 and proposed that this enhancement of neutralization was due to increased electrostatic interaction between MAbs and Env [49]. Furthermore, several of these mutations have been associated with changes in neutralization profiles. For example, the removal of N160 is responsible for resistance to PG9 and PG16 [50] but the addition of N160 correlates with increased neutralization resistance in some isolates [51]. A281V mutation also increases resistance to neutralization [52,53]. The late variants d417 D15, d487 A6-1 and d670 A8-9 have the R315K mutation and are resistant to 447-52D (data not shown). Similarly, primary viruses 5768-27p and QH0515 have the same R315K mutation [42] and are more resistant to neutralization by 447-52D [54,55].

Finally, our data show that the mutations and combinations that conferred 2G12 resistance are subject-specific. One caveat is that our findings result from examination of a single animal and we speculate that other PNGS may be discovered in additional SHIV-infected macaques. Indeed, different combinations of PNGS and amino acid changes from A141 were associated with 2G12 resistance in Envelopes from clade B HIV-infected subjects [23,24]. We hypothesize that the 2G12 resistance in subjects ACH19545, ACH11686 and ACH18860 is most likely due to the absence of one or several accessory PNGS (339,386,397) for 2G12 recognition [19,34]. Accessory PNGS can be altered without eliminating 2G12 binding. Indeed, due to the large footprint of glycans, a previous study showed that 2G12 binds to a fraction of identified PNGS and, in the context of natural sequences, some of the identified glycans can be absent without compromising 2G12 binding [34]. Subject ACH18839, like A141, has all required and accessory PNGS for 2G12 interaction [34] but most resistant ACH18839 clones have an additional PNGS at position 406. Given that PNGS N406 is present in all A141 Envelopes we speculate that this glycan may be responsible for 2G12 resistance in a context-dependent manner as previously shown for PNGS N386 and N302 [21,56]. Furthermore, we identified amino acid changes at different positions in ACH18839 and A141 (Tables 1 and 2) but like in A141 they introduced more negative charges that could affect envelope conformation and antibody recognition [13]. Additional Env changes affecting 2G12 neutralization were previously identified including a PNGS addition at position 302 [21] and mutations T283N [22], D113A, D180A, S365A and N276A [57]. However, we identified only two of these changes here and only in a few Envs. The T283N mutation occurred in only one Env (ACH18839. R4.15H6, data not shown). The deletion of PNGS N276 occurred in two Envs (ACH19545. D1.6F3 and ACH18860. R1.6F5), but did not increase 2G12 sensitivity as previously reported [57].

In conclusion, our results and those of others [21,22,56] demonstrate that escape from 2G12 can be achieved through different pathways in a subject-specific manner, previously shown for escape from autologous pressure [5,6] and other MAbs [58,59], and no obvious common signature motifs could be identified [60] thus suggesting that this complex epitope may be a difficult target for vaccines. A focus on shared neutralization epitopes that remain stable under immune pressure may lead to more effective vaccines. 
